# Equilibrium Skyrmion Lattice Ground State in a Polar Easy-plane Magnet

**DOI:** 10.1038/s41598-017-07996-x

**Published:** 2017-08-08

**Authors:** S. Bordács, A. Butykai, B. G. Szigeti, J. S. White, R. Cubitt, A. O. Leonov, S. Widmann, D. Ehlers, H.-A. Krug von Nidda, V. Tsurkan, A. Loidl, I. Kézsmárki

**Affiliations:** 1 Department of Physics, Budapest University of Technology and Economics and MTA-BME Lendület Magneto-optical Spectroscopy Research Group, 1111 Budapest, Hungary; 2 Laboratory for Neutron Scattering and Imaging, Paul Scherrer Institut, CH-5232 Villigen, Switzerland; 3 Institut Laue-Langevin, 6 rue Jules Horowitz, 38042 Grenoble, France; 4 Center for Chiral Science, Hiroshima University, Higashi-Hiroshima, Hiroshima, 739-8526 Japan; 5 Department of Chemistry, Faculty of Science, Hiroshima University Kagamiyama, Higashi Hiroshima, Hiroshima, 739-8526 Japan; 6 Experimental Physics V, Center for Electronic Correlations and Magnetism, University of Augsburg, 86135 Augsburg, Germany; 7 Institute of Applied Physics, Academy of Sciences of Moldova, MD 2028 Chisinau, Republic of Moldova

## Abstract

The skyrmion lattice state (SkL), a crystal built of mesoscopic spin vortices, gains its stability via thermal fluctuations in all bulk skyrmion host materials known to date. Therefore, its existence is limited to a narrow temperature region below the paramagnetic state. This stability range can drastically increase in systems with restricted geometries, such as thin films, interfaces and nanowires. Thermal quenching can also promote the SkL as a metastable state over extended temperature ranges. Here, we demonstrate more generally that a proper choice of material parameters alone guarantees the thermodynamic stability of the SkL over the full temperature range below the paramagnetic state down to zero kelvin. We found that GaV_4_Se_8_, a polar magnet with easy-plane anisotropy, hosts a robust Néel-type SkL even in its ground state. Our supporting theory confirms that polar magnets with weak uniaxial anisotropy are ideal candidates to realize SkLs with wide stability ranges.

## Introduction

Whether skyrmions, as nanometric bits^1, 2^, can boost the information density of magnetic memory devices depends on three key factors: i) their thermal stability range^3–11^, ii) size^12–14^ and iii) controllability by external stimuli, preferably via electric fields or weak electric currents^7, 15–19^. Some of the currently existing host materials at least partially fulfill these conditions and may reach the level of applications. As a common feature, all of them lack spatial inversion symmetry. When inversion symmetry is broken in a material with predominantly ferromagnetic interactions, the relativistic Dzyaloshinskii-Moriya interaction (DMI) emerges that can destabilize the ferromagnetic state (FM), giving rise to the formation of spin spirals and skyrmions^20–26^. Additionally, SkL-like phases may also exist in centrosymmetric magnets as a consequence of frustrated magnetic interactions^27, 28^, though this theoretical prediction has not found experimental realization yet.

In multilayer systems, consisting alternating magnetic and heavy metal layers, the inversion symmetry is broken by the interfaces and strong DMIs emerge in the magnetic layers due to their proximity to the heavy element layers. Recently, in such Pt/CoFeB/MgO, Pt/Co/Ta and Pt/Co/MgO stacks the stabilization of mid-sized skyrmions (typically 100 nm in diameter) has been demonstrated at room temperature^5–7^. The same interface-induced DMI mechanism leads to the spontaneous formation of SkL in iron mono-, bi- and triple layers with unprecedently small atomic-scale skyrmions at low temperatures^13, 14, 16–18^. Room-temperature SkLs with periodicities of ~100 nm were also reported to emerge in ultrathin films of FeGe^4^ and in bulk crystals of *β*-Mn-type Co-Zn-Mn alloys^8, 9^. In these cubic magnets, the spatial inversion symmetry is broken by the chirality of the lattice, similarly to the case of MnSi^3, 29, 30^ and its isostructural analogues^31–34^. In terms of current-driven skyrmion manipulation, a possible drawback of multilayers and Co-Zn-Mn compounds is the strong pinning of skyrmions due to atomic-scale disorder inevitably present in these systems.

Of the most desirable functionalities, the manipulation, writing and erasing of skyrmions solely by electric fields, i.e. without electric currents, have been demonstrated only in iron triple layers so far^17^. The possibility of such electric-field-driven switching was also proposed in magnetoelectric Cu_2_OSeO_3_^35–37^ and in multiferroic lacunar spinel GaV_4_S_8_, a polar magnet hosting Néel-type skyrmions^38, 39^ that are dressed with local electric polarization^40, 41^. Besides the strong coupling between its magnetic pattern and the local electric polarization, the orientational confinement is another peculiarity of this new type of skyrmions. Their cores remain rigidly aligned with the polar axis of the crystal even in oblique magnetic fields^38, 39^, in strong contrast with Bloch-type skyrmions in cubic chiral magnets where the cores are always aligned with the field. The unique pattern of DMI vectors, dictated by the polar C_*nv*_ symmetry^21, 24, 26^, is responsible for this orientational confinement and may provide a route towards an enhanced stability of the SkL relative to SkLs in cubic chiral magnets. In GaV_4_S_8_ the uniaxial anisotropy is relatively strong and of easy-axis type^42, 43^, ultimately overcoming the DMI and enforcing the ferromagnetic order in the ground state^38^. Nevertheless, the cycloidal and SkL states are stabilized via thermal fluctuations over a broader range of *T*/*T*_*C*_, with *T*_*C*_ being the magnetic ordering temperature, than found in any other bulk skyrmion-host materials so far. This makes magnets with C_*nv*_ symmetry promising candidates to host bulk SkLs with broad stability ranges.

Here we study the magnetic phases of GaV_4_Se_8_, another member of the rich family of AM_4_X_8_-type (A = Ga or Ge; M = Mo, V or Nb and X = S or Se) lacunar spinels. Many of these compounds with M = Mo and V undergo a Jahn-Teller transition from a room-temperature cubic structure (point group T_*d*_) into a low-temperature rhombohedral polar structure (C_3*v*_)^40, 44–47^. Lacunar spinels are molecular magnets with M_4_ clusters as building blocks, which carry an effective S = 1/2 spin in case of M = V and Mo^44^. In the rhombohedral state, the M_4_ tetrahedra are distorted along one of the four cubic 〈111〉 axes, lowering the point group symmetry of individual tetrahedra as well as the whole crystal to C_3*v*_ (see later in Fig. 3a). Within the polar state these compounds undergo a long-range magnetic ordering and hence they become type-I multiferroics^40, 47^. In GaV_4_Se_8_ the structural transition takes place at T_*s*_ = 42 K and the magnetic ordering occurs at T_*C*_ = 18 K^40^. This polar distortion leads to a special DMI pattern, the prerequisite of Néel-type SkLs^21, 24, 26, 38^, and a uniaxial magnetic anisotropy^42, 43^. A similar DMI pattern is realized in ultra thin magnetic layers and multilayers near the interfaces^5, 6, 10, 13, 19^. Very recently, Fujima and coworkers have indeed reported the possible emergence of modulated magnetic phases in GaV_4_Se_8_^48^. They found low-field anomalies in the magnetization and magnetocurrent curves below 19 K what they interpreted as transitions between a cycloidal, a Néel-type SkL and a field polarized FM state, in analogy with GaV_4_S_8_^38^.

## Results

### Axial symmetry, polar structure and domains

**Figure 1:**
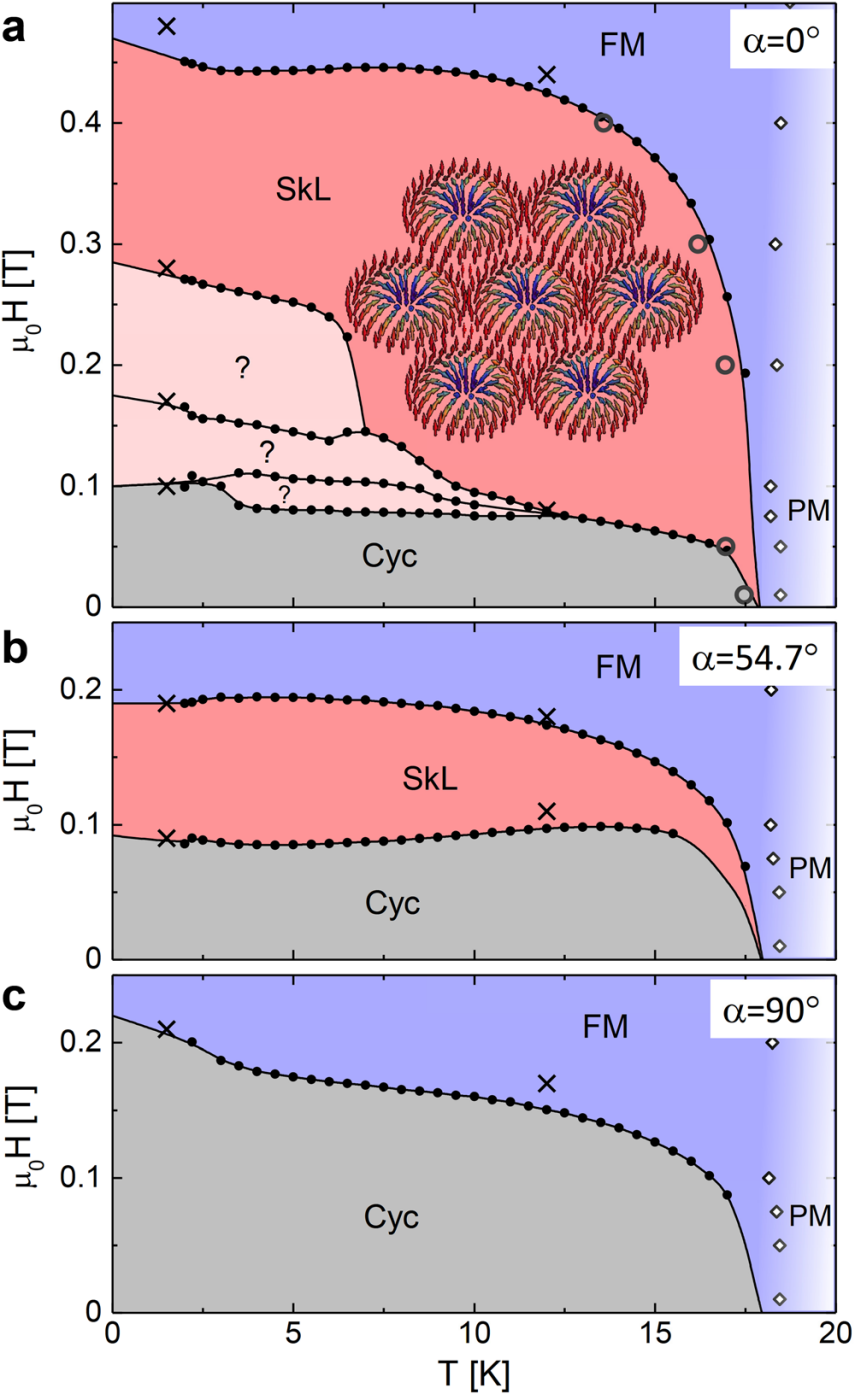
Extended cycloidal and Néel-type SkL states in GaV_4_Se_8_. (**a**) Magnetic phase diagram for magnetic fields parallel to the polar axis, *α* = 0°. Below T_*C*_ = 18 K, the cycloidal (Cyc) and SkL phases, underlying the field polarized ferromagnetic state (FM), persist down to the lowest temperatures. The Néel-type SkL spin texture is schematically illustrated. Phase boundaries are assigned to anomalies in ∂*M*/∂*H* versus *H* (closed circles) and ∂*M*/∂*T* versus *T* (open circles) curves and by the SANS data (crosses). Lines are guides to the eye. The crossover region between the paramagnetic (PM) and FM states is indicated by open diamonds and color gradation. Below 12 K additional phases, labeled by question marks, emerge between the cycloidal and SkL states. (**b**) Magnetic phase diagram for fields applied at *α* = 54.7° with respect to the polar axis. In the phase labeled as Cyc, the magnetic field component normal to the polar axis continuously distorts the cycloid into a tilted conical structure, as visualized in Fig. 4g. The field component along the polar axis still establishes the SkL before the FM is reached. (**c**) For magnetic fields applied perpendicular to the polar axis, *α* = 90°, the Cyc state corresponds to the regular cycloid, which is smoothly canted by the field to form a transverse conical structure, as sketched in Fig. 4e, and eventually transforms into the FM.

Our electron spin resonance (ESR) spectroscopy results, described in the following, show that GaV_4_Se_8_ is characterized by a weak easy-plane type axial anisotropy, in contrast to GaV_4_S_8_ with easy-axis anisotropy. As we will show, this dramatically enhances the stability range of the SkL both in terms of temperature and magnetic field. Indeed, our key finding is that the SkL remains stable down to zero kelvin and for magnetic fields applied along the polar axis up to ~450 mT, as shown in Fig. 1a. Moreover, the SkL persists in oblique fields, as in Fig. 1b, where the magnetic field spans 54.7° with the polar axis.

As for GaV_4_S_8_^38, 41^, the structural transition leads to a multi-domain rhombohedral state, with all four rhombohedral twins present in the material. In terms of magnetic properties this means the coexistence of domains with four different anisotropy axes, namely [111], $\mathrm{[1}\overline{11}]$, $[\overline{1}1\overline{1}]$, $[\overline{11}\mathrm{1]}$. If the external magnetic field spans different angles with the anisotropy axes, different sequences of magnetic phase transitions are expected in the non-equivalent domains^38^. In crystals with polar C_*nv*_ symmetry, the modulated magnetic patterns realized at zero field are spin cycloids, where the modulation vectors (q-vectors) favoured by the DMI are perpendicular to the polar axis, and the plane of each cycloid is spanned by its q-vector and the polar axis^22, 24, 25, 38^, as shown in Fig. 4d. For magnetic fields applied parallel to the polar axis, at a critical field value the spin cycloid transforms into a Néel-type SkL with the plane of the SkL being perpendicular to the polar axis^22, 24, 25, 38^. At a second critical field, the SkL transforms into the homogeneous FM. In contrast, when the magnetic field is perpendicular to the polar axis, the spin cycloid is continuously distorted into a transverse conical structure. The modulated component of the magnetization still rotates in a plane containing the polar axis but the additional uniform component, corresponding to the axis of the cone, is parallel to the external field (see Fig. 4e). At a critical field value the cone angle closes and the FM is formed without the emergence of an intermediate SkL^42^.

In the following, we characterize the magnetic anisotropy of GaV_4_Se_8_ by ESR spectroscopy and its magnetic phase boundaries using magnetization and small angle neutron scattering (SANS) experiments. The modulated magnetic textures in the different phases are assigned based on the SANS data and the comparison between experimental and theoretical phase diagrams.

### Electron spin resonance spectroscopy

**Figure 2:**
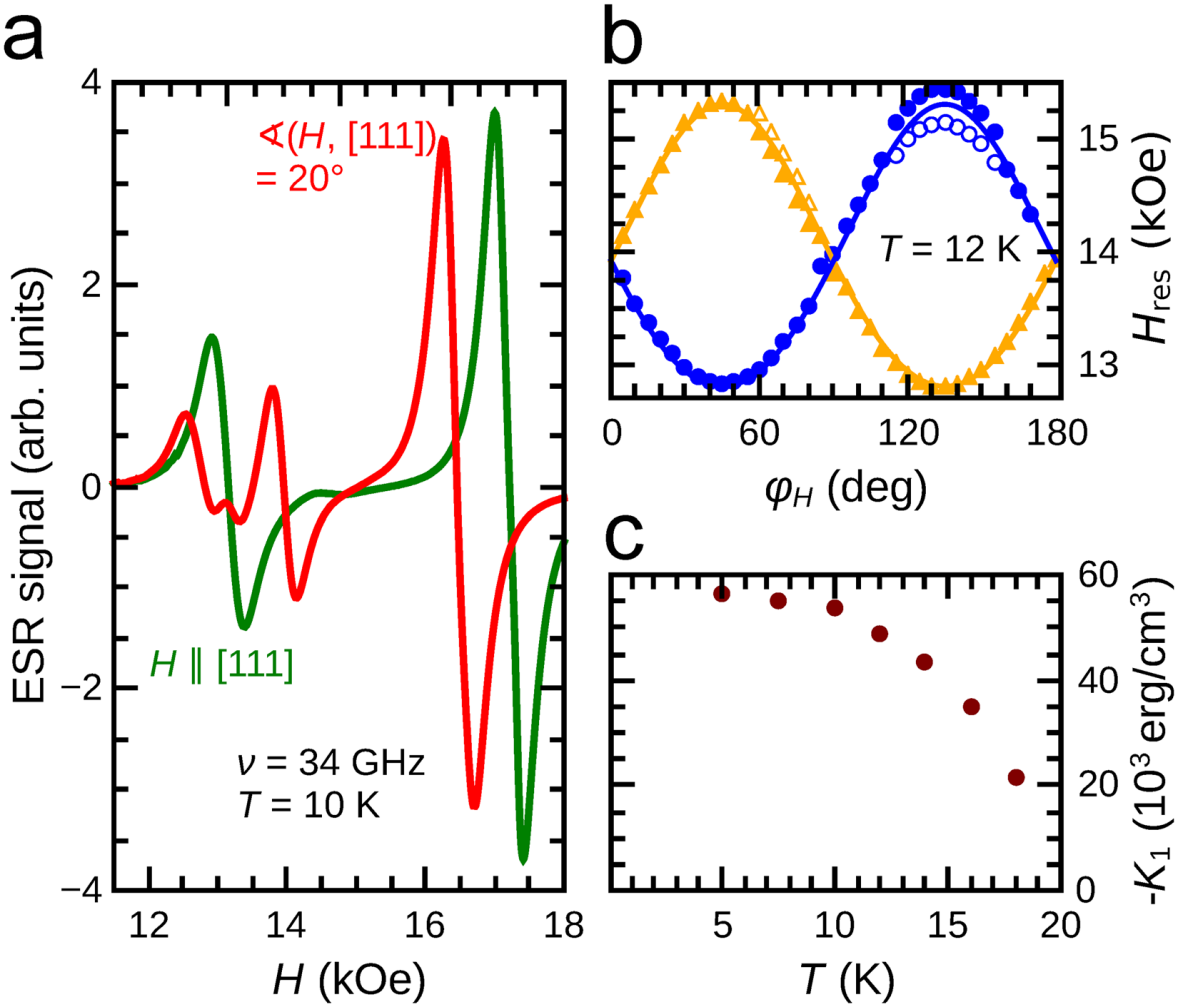
Magnetic anisotropy of GaV_4_Se_8_. (**a**) Electron spin resonance spectra for the external field *H* applied along a rhombohedral axis (green curve) and tilted by 20° away from it (red curve). (**b**) Resonance fields (symbols) for the magnetic field rotated within the (001) plane, where *φ*_*H*_ is the angle between the field and the [001] direction, i.e. 0°, 45°, 90° and 135° correspond to the [100], [110], [010] and [1$\overline{1}$0] axes, respectively. Solid lines display a fit based on uniaxial anisotropy. (**c**) Temperature evolution of the anisotropy constant *K*_1_ < 0, characteristic of easy-plane anisotropy.

The magnetocrystalline anisotropy of GaV_4_Se_8_ was determined by ESR spectroscopy upon rotation of the magnetic field within the (001) plane. All the ESR experiments were carried out in the field polarized FM state of the compound. In this case a single magnetic resonance is expected for each structural domain, with a resonance field depending on the relative orientation of the magnetic field and the rhombohedral axis of the given domain. At 12 K, the angular dependence of resonance fields, as extracted from Lorentzian derivative fits to the spectra, is depicted in Fig. 2b. Two main resonances are followed when rotating the sample by 180°. Assuming uniaxial anisotropy along the four 〈111〉 axes, these two branches are understood as belonging to two pairs of rhombohedral domains, where each pair consists of two indistinguishable (magnetically equivalent) domains, as long as the external field is within the (001) plane. The splitting is largest, when the external field is applied along the [110] and [1$\overline{1}$0] axes. The splitting is zero for field applied along the [100] and [010] axes. In this case all four domains are magnetically equivalent. Both resonances exhibit minor splittings into subcomponents. The overall splitting is ~250 mT, significantly smaller than in GaV_4_S_8_, where the splitting was ~1 T^43^. The angular dependence of the resonance for both pairs of domains was fitted by a model with uniaxial magnetic anisotropy, using the Landé *g*-factor and first anisotropy constant *K*_1_ as free parameters. The *g*-factor was found to be *g* = 1.71, independent of temperature, which is comparable to the value found in GaV_4_S_8_^43^. The temperature evolution of *K*_1_ is shown in Fig. 2c, where a strong decrease is observed upon approaching the Curie temperature.

The sign of the anisotropy constant, distinguishing between easy-plane and easy-axis anisotropies, is unraveled by additional measurements, when the magnetic field was applied along the [111] axis. In this case we again expect two resonances: one coming from the unique domain whose rhombohedral axis is parallel to the field and another from the three domains whose rhombohedral axes span 70.5° with the field. Indeed, the corresponding spectrum has two resonances, as shown in Fig. 2a. To determine which resonance corresponds to which set of domains, we also measured the ESR spectrum with the field rotated by 20° away from the [111] rhombohedral axis in an arbitrary plane. Since the resonance at higher fields is not split by the rotation of the field to an arbitrary direction, while the lower-field one is split into three components indicates that the former comes from the unique domain and the other originates from the other three domains.

The important finding is that–among all orientations of the magnetic field–the maximal resonance field is observed when the field is applied along the rhombohedral axis, which is the situation for an easy-plane magnet, and thus *K*_1_ must be negative. This experimental finding contradicts the presence of easy-axis type magnetic anisotropy in GaV_4_Se_8_, assumed by Fujima and coworkers^48^.

### Magnetic phase diagrams

**Figure 3:**
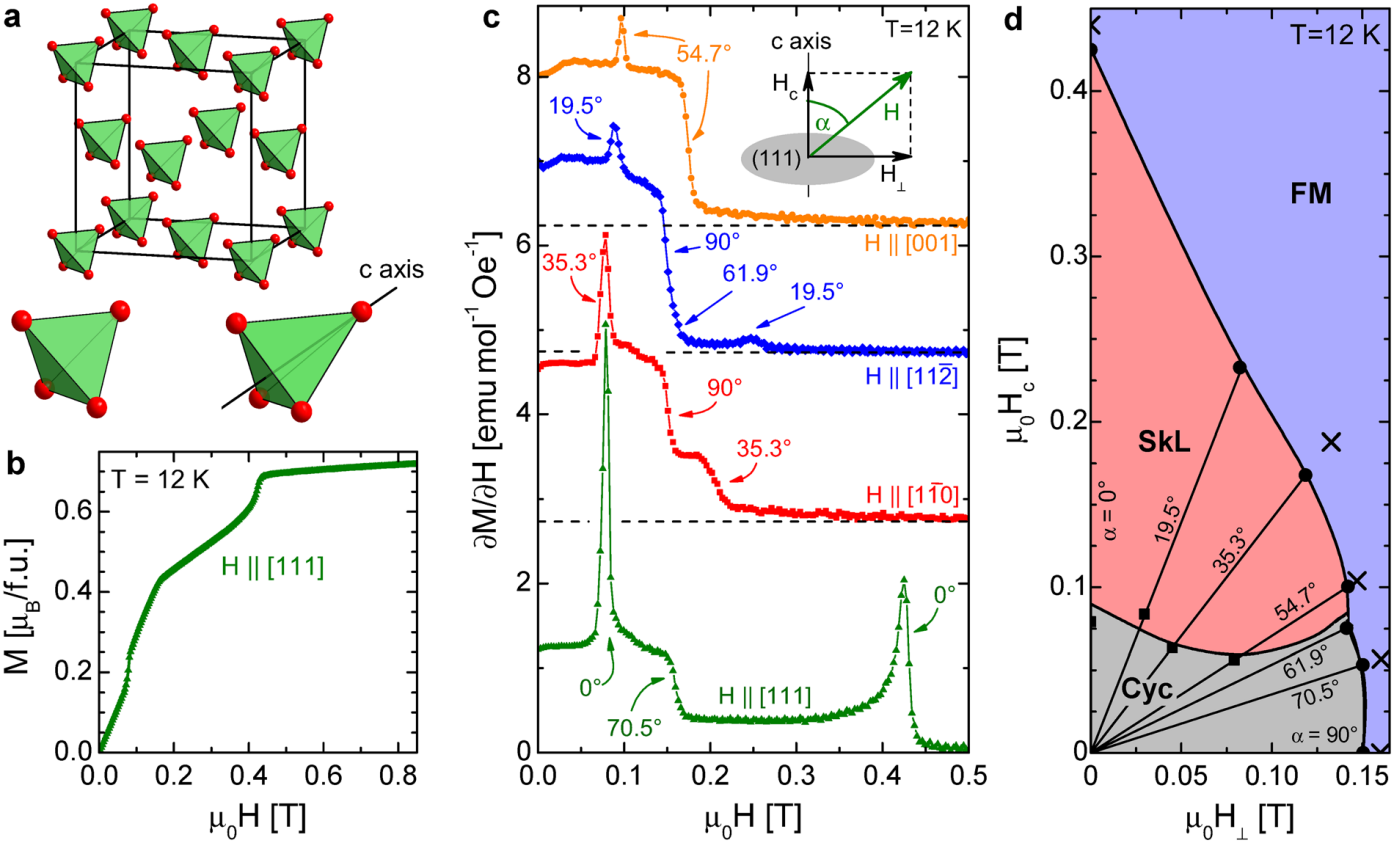
Structural and magnetic properties of GaV_4_Se_8_. (**a**) Crystal structure of GaV_4_Se_8_. For the sake of clarity only the face centered cubic lattice of V_4_ tetrahedra is shown. The V_4_ building block is shown both in the cubic state (T_*d*_) and the rhombohedral state (C_3*v*_), when the crystal is stretched along a 〈111〉-type axis, labeled as the *c* axis. (**b**) Field dependence of the magnetization at 12 K. (**c**) ∂*M*/∂*H* versus H curves measured at 12 K for **H** ‖ [111], [1$\overline{1}$0], [11$\overline{2}$], and[001]. The curves are vertically shifted relative to each other. Anomalies are labeled with the angles *α* spanned by the magnetic field and the polar *c* axes of the domains wherein the corresponding phase transitions take place. (**d**) Stability regions of the cycloidal (Cyc), SkL and ferromagnetic (FM) states at 12 K on the H_*c*_–H_⊥_ plane, where H_*c*_ and H_⊥_ are the field components along and perpendicular to the polar *c* axis. Phase boundaries are assigned by anomalies observed in the ∂*M*/∂*H* curves in panel (c) (full symbols) and by the SANS data (crosses) for various *α* angles. Lines connecting full symbols are guides to the eye. In finite H_⊥_ the cycloidal state is distorted to transverse or tilted conical structures as shown in Fig. 4e,g.

Figure 1 displays magnetic phase diagrams determined on the basis of magnetization and SANS experiments. The M(H) magnetization curve in Fig. 3b, representative for magnetic fields (H) applied along any of the four cubic 〈111〉 axes, shows three distinct anomalies at T = 12 K. For **H** ‖ [111], the field is parallel to the magnetic hard axis (polar axis) of one type of rhombohedral domain, while it spans ~70.5° with the hard axes of the other three types of domains. In the rhombohedral setting the polar axis is referred to as the *c* axis. We assign the lowest and highest field anomalies, taking place at 80 mT and 440 mT, respectively as the signatures of the cycloidal to SkL and the SkL to ferromagnetic transitions taking place in the unique domain. The third anomaly at 160 mT indicates a magnetic transition in the other three domains. Since in these domains the magnetic field is nearly perpendicular to the polar axes, this transition corresponds to the closing of the cone angle, i.e. the transition from the transverse conical to the FM state. These metamagnetic transitions show up even more clearly in the corresponding differential susceptibility curve in Fig. 3c. By following the temperature dependence of the anomalies (see the Supplementary Information), the phase boundaries separating the cycloidal, SkL and FM are determined and displayed in Fig. 1a. Below 12 K additional phases emerge between the cycloidal and SkL states, whose nature will be discussed later.

**Figure 4:**
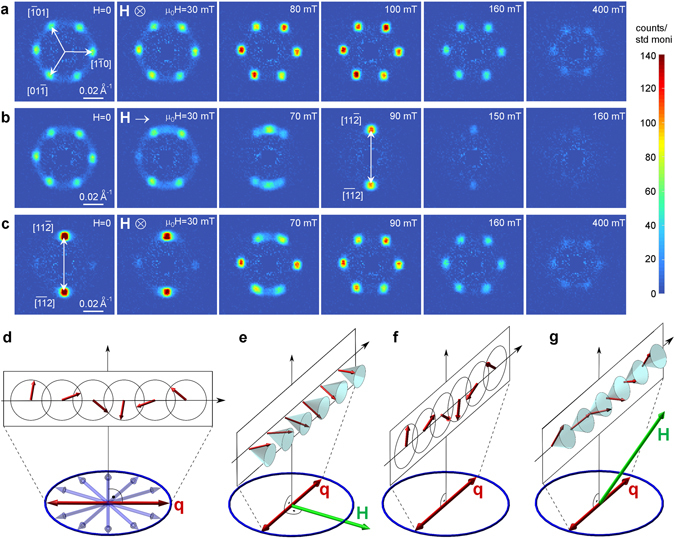
Modulated magnetic structures in GaV_4_Se_8_ at 12 K. (**a**) Representative SANS images recorded in the (111) plane with magnetic field applied along the [111] axis. The first image was taken after zero-field cooling. The magnetic field increases from left to right. (**b**) The same as for panel (a) but with field applied along the [1$\overline{1}$0] axis. (**c**) After the measurements in ${\bf{H}}\parallel [1\overline{1}\mathrm{0]}$, the magnetic field was decreased to zero and SANS images were again recorded in the (111) plane with **H** ‖ [111]. The colour scale on the right is common for panels (a–c). (**d**) Schematic of cycloidal structures present with arbitrary q-vectors distributed around a ring in the rhombohedral plane. For clarity, the cycloid is shown only for a single q-vector. (**e**) Magnetic fields applied perpendicular to the polar axis establish a transverse conical state with single q-vector normal to the field. (**f**) After switching off the magnetic field, this conical structure relaxes back to a cycloid preserving the single q-vector state. (**g**) For **H** with oblique angles, the field component normal to the polar axis distorts the cycloid into a tilted conical structure, where the axes of the cones are tilted away from the polar axis but are not exactly parallel with the field.

The magnetization was measured for three other orientations of the magnetic field, namely for ${\bf{H}}\parallel [11\overline{2}]$, $\mathrm{[1}\overline{1}\mathrm{0]}$ and [001]. The differential susceptibility curves measured at 12 K for these three orientations are also plotted in Fig. 3c. The angles *α* are those spanned by the magnetic field and the polar axes of the domains in which the corresponding phase transitions occur. When ${\bf{H}}\parallel \mathrm{[11}\overline{2}]$, the magnetic field is perpendicular to the polar axes of [111]-type domains, while for ${\bf{H}}\parallel [1\overline{1}\mathrm{0]}$ the field is orthogonal to the polar axes of both [111]- and  $[\overline{11}\mathrm{1]}$-type domains. The step-like anomalies observed in both cases in the differential susceptibility curves at 150 mT signal the transition from the transverse conical structure to the FM. This critical field is slightly lower than 160 mT found for *α* = 70.5°. By further decreasing the angle between the field and the polar axis to *α* = 61.9°, we still observe the direct transformation of the tilted conical state to the FM, likely due to the larger transverse susceptibility of this state as compared to that of the SkL. However, for *α* = 54.7° the intermediate SkL already emerges and its field stability range continuously extends with decreasing angle, as traced for *α* = 35.5°, 19.5° and 0°. These results are summarized in Fig. 3d, where the stability regions of the different phases at 12 K are shown on the *H*_⊥_–*H*_*c*_ plane, with *H*_*c*_ and *H*_⊥_ being the magnetic field components parallel and perpendicular to the polar axis, respectively. For illustrations of the cycloidal, tilted and transverse conical structures see Fig. 4d–g.

The thermal stability ranges of the different phases are also shown for *α* = 54.7° in Fig. 1b and for *α* = 90° in Fig. 1c. For **H** ‖ [001], all types of domains are magnetically equivalent with *α* = 54.7° common for each of them. As compared with *α* = 0°, at this oblique angle the cycloidal state—more precisely, the tilted conical state depicted in Fig. 4g—extends up to higher fields and the field stability range of the SkL is reduced. Nevertheless, both phases are stable down to the lowest temperatures. For *α* = 90° the transverse conical state, directly transforming to the ferromagnetic state, remains stable over the whole temperature region below T_*C*_.

### Assignment of magnetic phases

SANS experiments were performed at 12 K and 1.5 K with different orientations of the magnetic field. The two main purposes of the SANS study were i) to directly distinguish which magnetic anomalies originate from which types of structural domains, i.e. to confirm the assignment of the phases and *α* angles in Figs 1 and 3 and ii) to determine the periodicity of both the spin cycloid (transverse cone) and the SkL. Figure 4 shows a collection of SANS images with the incoming neutron beam parallel to the [111] axis. Correspondingly, magnetic scattering was recorded in the (111) plane. Note that due to the multi-domain crystal state the SANS intensity has contributions from all four types of rhombohedral domains. In the present case, the (111) image plane contains all information about the domains with the [111] rhombohedral axis, since the possible magnetic q-vectors in this type of domain are nearly confined to the (111) rhombohedral plane by the axial symmetry and the corresponding DMI pattern of the compound^38^. For the other three types of domains the rhombohedral planes intersect the (111) image plane along the three $\langle 1\overline{1}0\rangle$-type axes, thus, magnetic scattering from those domains can only be detected along these directions.

**Figure 5:**
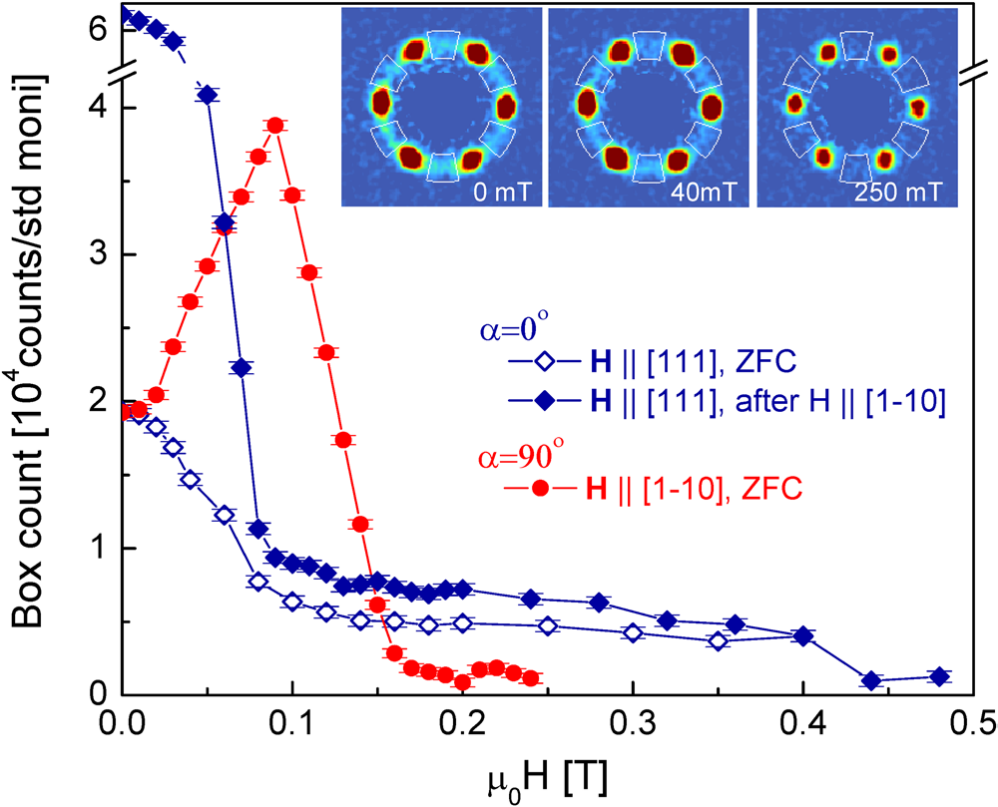
SANS intensity due to a single rhombohedral domain. Field dependence of the SANS intensity measured for selected parts of the ring structure within the white sectors, as shown in the SANS images recorded in 0, 40 and 250 mT with **H** ‖ [111]. The three curves correspond to the three measurement configurations presented in Fig. 4a–c.

SANS images in Fig. 4a summarize the field dependence of the scattered intensity at *T* = 12 K with **H** ‖ [111]. For each field value, a hexagonal pattern is observed with six $\langle 1\overline{1}0\rangle$-type q-vectors. Both in the cycloidal and SkL states the same hexagonal pattern is expected because of both the structural and magnetic multi-domain nature of the material^38, 41^. It is important to note that additional magnetic scattering is detected over a ring connecting the six spots, which exclusively comes from the domain with [111] rhombohedral axis. By analyzing the scattered intensity within the ring structure, we gain information selectively about the magnetic states in the unique domain with [111] rhombohedral axis. As also reported for GaV_4_S_8_^49^, this ring indicates that the magnetic q-vectors are not strictly confined to $\langle 1\overline{1}0\rangle$-type directions but are freely distributed along any direction within the rhombohedral plane. A faint ring structure is even observed up to 400 mT, i.e. the close vicinity of the FM, as analyzed in detail in Fig. 5. This implies that not only the cycloids can possess any q-vector around the ring but that the SkL may also exhibit a significant degree of in-plane orientational disorder. The cycloidal pitch and the lattice constant of the SkL are found to be *λ*_*cyc*_ = 2*π*/*q* ≈ 19.4 nm and a_*SkL*_ = $4\pi /\sqrt{3}q$ ≈ 22.4 nm, respectively. These values are nearly independent of temperature and magnetic field, except for an enhancement of a_*SkL*_ in the vicinity of the additional phases emerging for **H** ‖ [111] and close to the FM state.

For the case of SANS data shown in Fig. 4b, the magnetic field is applied along the $[1\overline{1}0]$ direction, i.e. perpendicular to the polar axis of the unique rhombohedral domain. With increasing field, the intensity originally distributed over the ring gets concentrated into two spots, **q** = ±$[11\overline{2}]$. Thus, in the unique domain the cycloidal state, originally realized with arbitrary q-vectors around the ring, is gradually transformed into a transverse conical state with well-defined **q** = ±$[11\overline{2}]$ and with the conical axis parallel to the field, as schematically sketched in Fig. 4d. At about 160 mT, the disappearance of the intensity from **q** = ±$[11\overline{2}]$ signifies the closing of the cone angle, in accord with Fig. 1c and the *α* = 90° cut of the phase diagram in Fig. 3d. In the other three structural domains, the magnetic field component perpendicular to their polar axes redistributes the intensity over similar rings in their rhombohedral planes to establish a uniform transverse or tilted conical state, shown in Fig. 4e,g, respectively. Therefore, the SANS intensity contributions coming from these three types of domains move out of the (111) image plane with increasing field and the magnetic states within these three types of domains cannot be further traced in this measurement geometry. More specifically, for $[\overline{11}\mathrm{1]}$-type domains, the field is also perpendicular to the polar axis, thus, the scattered intensity is redistributed around the ring in the $(\overline{11}1)$ plane. It is likely concentrated at **q** = ±[112], perpendicular to the field, upon the formation of a transverse conical state. For the remaining two types of domains with *α* = 35.3° the intensity moves to **q** = ±[110], accompanying the formation of tilted conical structures.

In Fig. 4c, SANS images were taken again with **H** ‖ [111]. However, in contrast to the zero-field cooling done before obtaining the data shown in Fig. 4a,b, these images were recorded right after the measurement shown in Fig. 4b, i.e. after the field ${\bf{H}}\parallel [1\overline{1}0]$ was reduced to zero. As is clear from the zero-field image of Fig. 4c, no change in the orientation of the q-vectors can be discerned after the removal of ${\bf{H}}\parallel [1\overline{1}0]$, hence a uniform cycloidal state persists in zero field (see Fig. 4f). The reapplication of **H** ‖ [111] causes a rearrangement of the q-vectors: The intensity originally concentrated at **q** = ±$[11\overline{2}]$ sharply drops and three pairs of ±$[1\overline{1}\mathrm{0]}$-type q-vectors gain intensity at around 80 mT, where the cycloidal to SkL transition is expected to take place. Indeed, the sudden rearrangement of the SANS intensity points toward the formation of the SkL state instead of the emergence of new cycloidal domains with different q-vectors, since no Zeeman energy gain is associated with the rotation of the cycloidal q-vector in the (111) plane, when the magnetic field is applied along the [111] axis. With increasing field, intensity contributions from the other domains also appear in the six $\langle 1\overline{1}0\rangle$-type spots. Similarly to Fig. 4a, the scattered intensity vanishes above 400 mT, where the onset of the SkL to ferromagnetic transition is expected.

To unambiguously determine the phase boundaries corresponding to the magnetic states within the domains with *α* = 0° and 90°, we analyze the intensity of the ring in the (111) image plane excluding regions with overlapping contributions from the other three types of domains, i.e. the vicinity of the six $\langle 1\overline{1}0\rangle$-type spots. In Fig. 5, the field dependence of the intensity summed over the rest of the ring, indicated by the white boxes in the insets, is shown for the three cases corresponding to Fig. 4a–c. For **H** ‖ [111] (*α* = 0°), there are two anomalies in the magnetic scattering intensity at 80 mT and 440 mT, the signatures of the cycloidal to SkL and the SkL to ferromagnetic transitions. The large difference in the low-field SANS intensity values below 80 mT is due to the difference in the history of the sample, zero-field cooling for Fig. 4a and field training in ${\bf{H}}\parallel [1\overline{1}0]$ for Fig. 4c. In case of *α* = 90°, the intensity summed over the restricted regions of the ring increases with increasing field, as expected from Fig. 4b, where the migration of the intensity from the whole area of the ring to **q** = ±$[11\overline{2}]$ is observed. After reaching a maximum, the intensity drops and vanishes at 160 mT, indicating that the transverse conical structure indeed turns to a homogeneous ferromagnet at this critical field. Additional SANS experiments, not presented here, were also carried out with the incoming neutron beam parallel to the $[1\overline{1}\mathrm{0]}$ and [100] axes. These results are fully consistent with the assignments of the different phases and *α* angles shown in Figs 1 and 3.

Here we briefly discuss our findings concerning the additional phases emerging for *α* = 0° below 12 K. Our SANS measurements performed at 1.5 K with **H** ‖ [111] clearly show that these extra phases between the cycloidal and the SkL states are present only for small *α*, more specifically present for *α* = 0° and already absent for *α* = 35.3°. These phases are manifested by anomalies in each of the magnetization, the SANS intensity and the length of the q-vectors. However, in the SANS images the hexagonal pattern of spots as well as the ring structure remain common features of the data obtained within all of these phases. Therefore, we can exclude the emergence of a square lattice of skyrmions recently proposed for such polar materials with easy-plane type axial anisotropy^25, 26, 50^. On the basis of systematic Monte-Carlo studies, the same authors proposed the emergence of an elliptical cone phase, which may be one of the extra phases observed in GaV_4_Se_8_. Fractionalization of skyrmions and emergence of additional exotic phases were also predicted for systems with axial anisotropy^50, 51^. The additional phases may also correspond to i) other distorted forms of the cycloidal state due the presence of lamella-like rhombohedral domain structures on the sub-micrometer scale or ii) further modulations developing along the polar axis due to frustrated exchange interactions. Future studies can aim to resolve the detailed microscopic properties of these phases.

### Supporting theory

Recently, Randeria and co-workers^26^ predicted the enhanced stability of SkLs in polar materials with Rashba-type spin-orbit interaction as compared to chiral compounds with Dresselhaus-type spin-orbit coupling. Their calculations also imply that easy-axis anisotropy suppresses the magnetic field stability range of the Néel-type SkL in the ground state of polar materials, while moderate easy-plane anisotropy, *A* < *D* ^2^/*J*, can progressively extend it. Here *D* and *J* stand for the strength of the DMI and the isotropic exchange interaction, respectively. These predictions are indeed in good agreement with the present finding of an extended SkL state in bulk GaV_4_Se_8_ with easy-plane anisotropy (see the Supplementary Information), and the suppressed stability range of the SkL in GaV_4_S_8_ with easy-axis anisotropy^38, 42, 43^.

In a Ginzburg-Landau approach, we address the impact of easy-plane anisotropy on the stability range of the cycloidal state and the Néel-type SkL in oblique magnetic fields, as studied experimentally in Figs 1 and 3d. The continuum free energy functional of a 2D polar ferromagnet with C_*nv*_ symmetry can be written as the sum of contributions from the exchange, DMI, Zeeman, and anisotropy energies:

1 $$w=\frac{J}{2}\sum _{i,j}\,{({\partial }_{i}{m}_{j})}^{2}+D({ {\mathcal L} }_{xz}^{(x)}+{ {\mathcal L} }_{yz}^{(y)})-{\bf{m}}\cdot {\bf{H}}+A{m}_{z}^{2},$$

where *x*, *y*, *z* coordinates are measured in units of the lattice constant and the magnetization is treated as a unit vector **m**. The magnetic field **H** spans an angle *α* with the polar *c* axis.

The Lifshitz invariants,

2 $${ {\mathcal L} }_{xz}^{(x)}+{ {\mathcal L} }_{yz}^{(y)}={m}_{x}{\partial }_{x}{m}_{z}-{m}_{z}{\partial }_{x}{m}_{x}+{m}_{y}{\partial }_{y}{m}_{z}-{m}_{z}{\partial }_{y}{m}_{y},$$

for C_3*v*_ symmetry promote modulated phases only with q-vectors perpendicular to the rhombohedral *c* axis, i.e. in the *xy*-plane of our model. Correspondingly, the longitudinal conical phase with q-vectors parallel to the magnetic field cannot be stabilized as discussed later. This is a key ingredient of the wide stability range of Néel-type SkLs in bulk materials, since the stability range of the Bloch-type SkLs in cubic chiral magnets is suppressed via its competition with the longitudinal conical state, which can be stabilized for arbitrary direction of the magnetic field. This is because a spin helix has the largest susceptibility along its q-vector, which thus co-aligns with the magnetic field to maximize the Zeeman energy term via the uniform magnetization component induced parallel to the field in addition to the magnetization component rotating perpendicular to the field. As predicted theoretically and confirmed experimentally^26, 50, 52–55^, axial anisotropy can also enhance the stability region of the Bloch-type SkL by narrowing the q-vectors into two dimensions.

**Figure 6:**
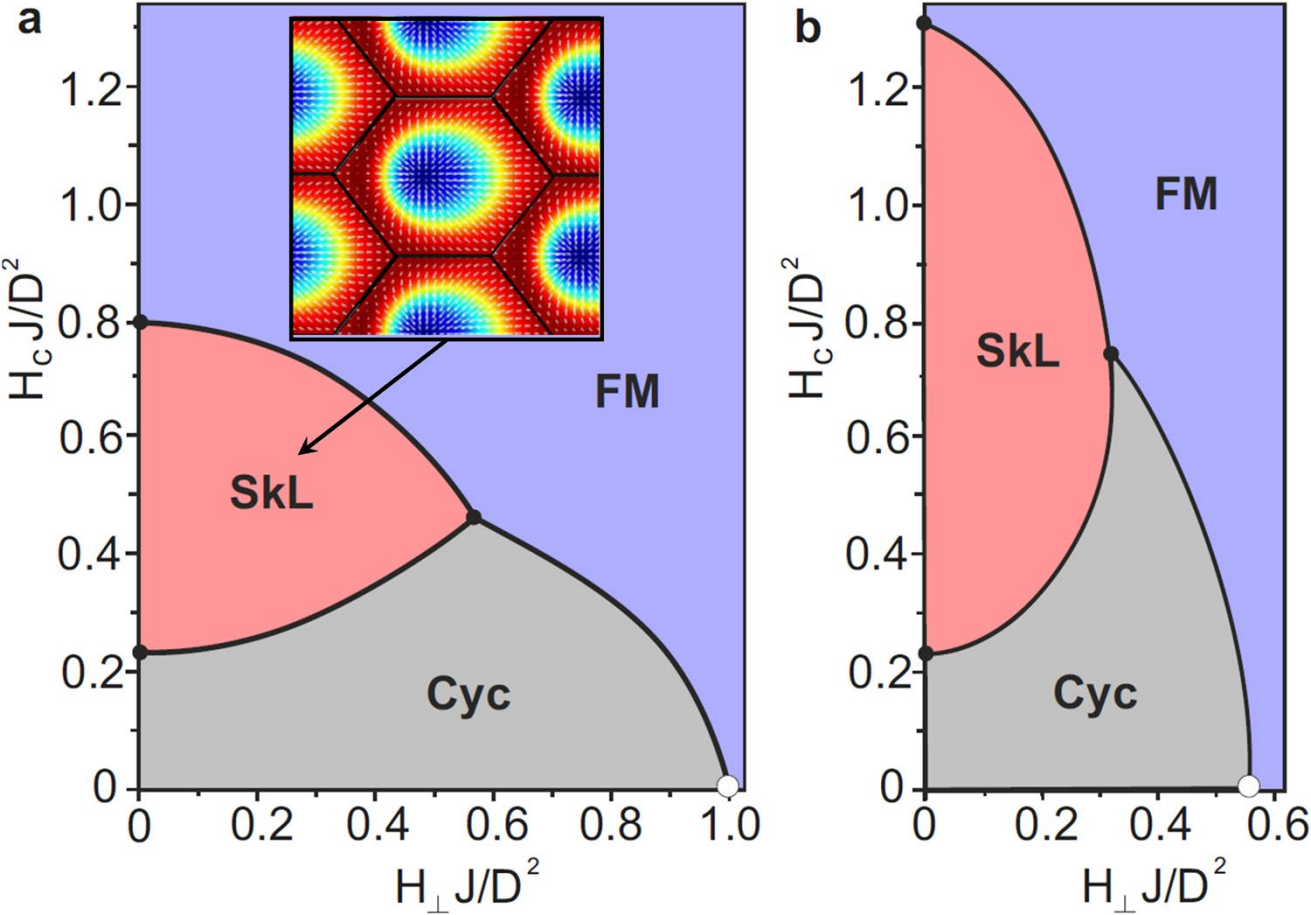
Angular stability of the Néel-type SkL. Stability regions of the cycloidal (Cyc), SkL and ferromagnetic (FM) states on the *H*_*c*_ *J*/*D* ^2^–*H*_⊥_ *J*/*D* ^2^ plane, where H_*c*_ and H_⊥_ are respectively the field components along and perpendicular to the polar *c* axis, as calculated for (**a**) *AJ*/*D* ^2^ = 0 and (**b**) *AJ*/*D* ^2^ = 0.5. The inset of panel (a) shows the distorted SkL with skyrmion cores displaced horizontally from the center of the unit cell along the *H*_⊥_ field component.

The robustness of the Néel-type SkL against oblique fields was studied for different values of the effective anisotropy parameter, *AJ*/*D* ^2^. Figure 6a,b respectively show the phase diagrams obtained for *AJ*/*D* ^2^ = 0 and *AJ*/*D* ^2^ = 0.5, these being representative of a system with C_*nv*_ symmetry, but with zero effective anisotropy (panel a), or with a moderate easy-plane anisotropy (panel b) as appropriate for GaV_4_Se_8_. The latter reproduces the main aspects of the experimental phase diagram shown in Fig. 3d: i) the ratio of the critical fields required to reach the FM for fields applied parallel and perpendicular to the polar *c* axis, ii) the extended stability range of the SkL for fields applied along the polar axis as compared with the *AJ*/*D* ^2^ = 0 case, iii) the suppression of the angular (*α*) stability range of the cycloidal and SkL states, i.e. a suppressed stability against fields perpendicular to the polar axis, in comparison with *AJ*/*D* ^2^ = 0.

When the field is applied along the polar axis, the rotation plane of the spins is preserved and the cycloid can only gain a finite magnetization by increasing its anharmonicity. For perpendicular fields, we find the so-called transverse conical state with uniform magnetization induced along the field and modulated components restricted to the perpendicular plane, as sketched in Fig. 4e. For oblique fields a general distorted cone structure is realized, referred to as the tilted conical state in Fig. 4g. Since the Lifshitz invariants (or equivalently the DMI pattern) characteristic to the polar C_3*v*_ symmetry confine the q-vectors to the rhombohedral plane, the plane of the SkL is also preserved in oblique magnetic fields, which can deform the SkL only by shifting the skyrmion cores from the center of the unit cell, as depicted in the inset of Fig. 6a.

## Discussion

The existence of isolated metastable skyrmions with long lifetime requires the vicinity of their equilibrium parent phase, thus, the broad stability range of the SkL is a prerequisite for skyrmion based memory devices. The possibility to stabilize the SkL in bulk materials over the whole temperature range below *T*_*C*_, which is demonstrated here for the polar magnetic semiconductor GaV_4_Se_8_, opens new perspectives in the field of skyrmion-based information technology called skyrmionics. The Néel-type SkL ground state is expected to be a common feature of axially symmetric polar materials with predominantly ferromagnetic exchange interactions. The pattern of DMI vectors, specific to the polar C_*nv*_ crystallographic class, forbids the existence of the longitudinal conical state. The complete suppression of this longitudinal conical state, being the main competitor of the SkL in chiral cubic magnets, plays a key role in the extended stability range of Néel-type skyrmions. The present study also corroborates that the broad stability region of the SkL requires the weakness of the uniaxial magnetic anisotropy, with a preference on the easy-plane type anisotropy. These conditions can be realized in crystals with weak axial deformations with respect to the cubic symmetry or even in compounds with strong axial distortions if the magnetic ions has no orbital moment. Cubic magnetic semiconductors close to a structural instability, such as Ge_1−*x*_Mn_*x*_Te^56, 57^, may be good candidates to realize SkLs being thermodynamically stable from elevated temperatures down to zero kelvin.

## Methods

### Sample synthesis and characterization

Single crystals of GaV_4_Se_8_ with typical mass of 1–30 mg were grown by the chemical vapour transport method using iodine as the transport agent. The samples were characterized by powder x-ray diffraction, specific heat and magnetization measurements. The crystallographic orientation of the samples was determined by x-ray Laue and neutron diffraction prior to the magnetization and SANS studies, respectively.

### Electron spin resonance spectroscopy

Continuous-wave ESR was performed at a frequency of 34 GHz (Q-band). A Bruker ELEXSYS E500 spectrometer was used and the resonator was a cylindrical Bruker ER 920 cavity. A helium gas-flow cryostat allowed a temperature stability of 0.1 K. To measure the angular dependence of the resonance field in the naturally grown (001) plane the sample was mounted with its growth plane normal to the rotation axis of a goniometer. The external magnetic field was swept in the range of 0–1.8 T. The spectra shown in Fig. 2a correspond to the first derivative of the absorbed microwave power due to the use of lock-in technique with magnetic field modulation.

### Magnetization measurements

The magnetization measurements were performed using an MPMS from Quantum Design. The field dependence of the magnetization was measured in increasing temperature steps of 0.5 K following an initial zero-field cooling to 2 K. The temperature dependence of the magnetization in constant fields was measured upon cooling.

### Small-angle neutron scattering

SANS was performed on a 10 mg single crystal sample of GaV_4_Se_8_ using the D33 and D11 instruments at the Institut Laue-Langevin (ILL), Grenoble, France. In a typical instrument configuration, neutrons of wavelength 5 Å were selected with a FWHM spread (Δ*λ*/*λ*) of 10%, and collimated over a distance of 5.3 m before reaching the sample. The scattered neutrons were collected by a two-dimensional pixel-detector placed 5 m behind the sample. The SANS measurements were done by both tilting and rotating the sample and the magnet together through angular ranges (rocking angles) that moved the magnetic diffraction peaks through the Ewald sphere. The SANS patterns presented in Fig. 5 were constructed by summing up the intensity measured through the whole range of rocking angles for each pixel of the detector. Similar measurements were taken at a temperature above *T*_*C*_ and subtracted from data obtained at lower temperatures, to leave just the signal due to magnetic scattering. Experiments were performed in two configurations, in magnetic fields applied parallel and perpendicular to the neutron beam using a split coil magnet.

### Theory

Our model describes a 2D lattice of classical spins with C_*nv*_ symmetry. The magnetic energy density in Eq. (1)–with contributions from isotropic exchange, DMI, magnetic anisotropy and Zeeman energies–has been minimized using the iterative simulated annealing procedure and a single-step Monte-Carlo dynamics with the Metropolis algorithm^58^. We imposed periodic boundary conditions and performed simulations for lattices of different sizes to check the stability of the numerical routine. The value of the effective anisotropy, *AJ*/*D* ^2^ = 0.5, was chosen to match the ratio of the two critical fields, corresponding to the Cyc-SkL and SkL-FM transitions, in the calculation and in the real material for *α* = 0. Differences between the theoretical and experimental phase diagrams, respectively displayed in Figs 3d and 6, may come from the following factors: i) unlike in our 2D model with uniform bonds, in the rhombohedral state GaV_4_Se_8_ has two set of bonds characterized by different exchange coupling, DMI and anisotropy, ii) for simplicity, in the axial magnetic anisotropy we only kept the ${m}_{z}^{2}$ term and neglected (∂_*x*_ *m*_*y*_)^2^ and (∂_*y*_ *m*_*x*_)^2^ terms, iii) due to additional magnetic states at low temperatures, the experimental magnetic phase diagram was constructed for 12 K (≈0.67 T_*C*_), where thermal fluctuations are still present. The thermal fluctuations, not captured by our model, are manifested in the reduced ordered moment observed in the FM state at 12 K as compared to 2 K.

### Data availability

The datasets generated during and/or analysed during the current study are available from the corresponding author on reasonable request.

## Electronic supplementary material

Supplementary information
