# Supplementary material for: Equilibrium Skyrmion Lattice Ground State in a Polar Easy-plane Magnet
Source: Sci Rep. 2017 Aug 8;7:7584. doi: 10.1038/s41598-017-07996-x (PMC5548730; doi:10.1038/s41598-017-07996-x)
Supplement: Supplementary file 1 — Supplementary information [file 41598_2017_7996_MOESM1_ESM.pdf]

Supplementary Information for Equilibrium Skyrmion Lattice Ground State in a Polar Easy-plane Magnet

S. Bordács,[1] A. Butykai,[1] B. G. Szigeti,[1] J. S. White,[2] R. Cubitt,[3] A. O. Leonov,[4,5] S. Widmann,[6] D. Ehlers,[6] H-A. Krug von Nidda,[6] V. Tsurkan,[6,7] A. Loidl,[6] and I. Kézsmárki[1,6]

[1]Department of Physics, Budapest University of Technology and Economics and MTA-BME Lendület Magneto-optical Spectroscopy Research Group, 1111 Budapest, Hungary
[2]Laboratory for Neutron Scattering and Imaging, Paul Scherrer Institut, CH-5232 Villigen, Switzerland
[3]Institut Laue-Langevin, 6 rue Jules Horowitz, 38042 Grenoble, France
[4]Center for Chiral Science, Hiroshima University, Higashi-Hiroshima, Hiroshima 739-8526, Japan
[5]Department of Chemistry, Faculty of Science, Hiroshima University Kagamiyama, Higashi Hiroshima, Hiroshima 739-8526, Japan
[6]Experimental Physics V, Center for Electronic Correlations and Magnetism, University of Augsburg, 86135 Augsburg, Germany
[7]Institute of Applied Physics, Academy of Sciences of Moldova, MD 2028, Chisinau, Republica Moldova

Magnetic phase diagrams. Figure 2 documents the determination of the phase boundaries based on magnetization experiments.

Typeset by REVTEX

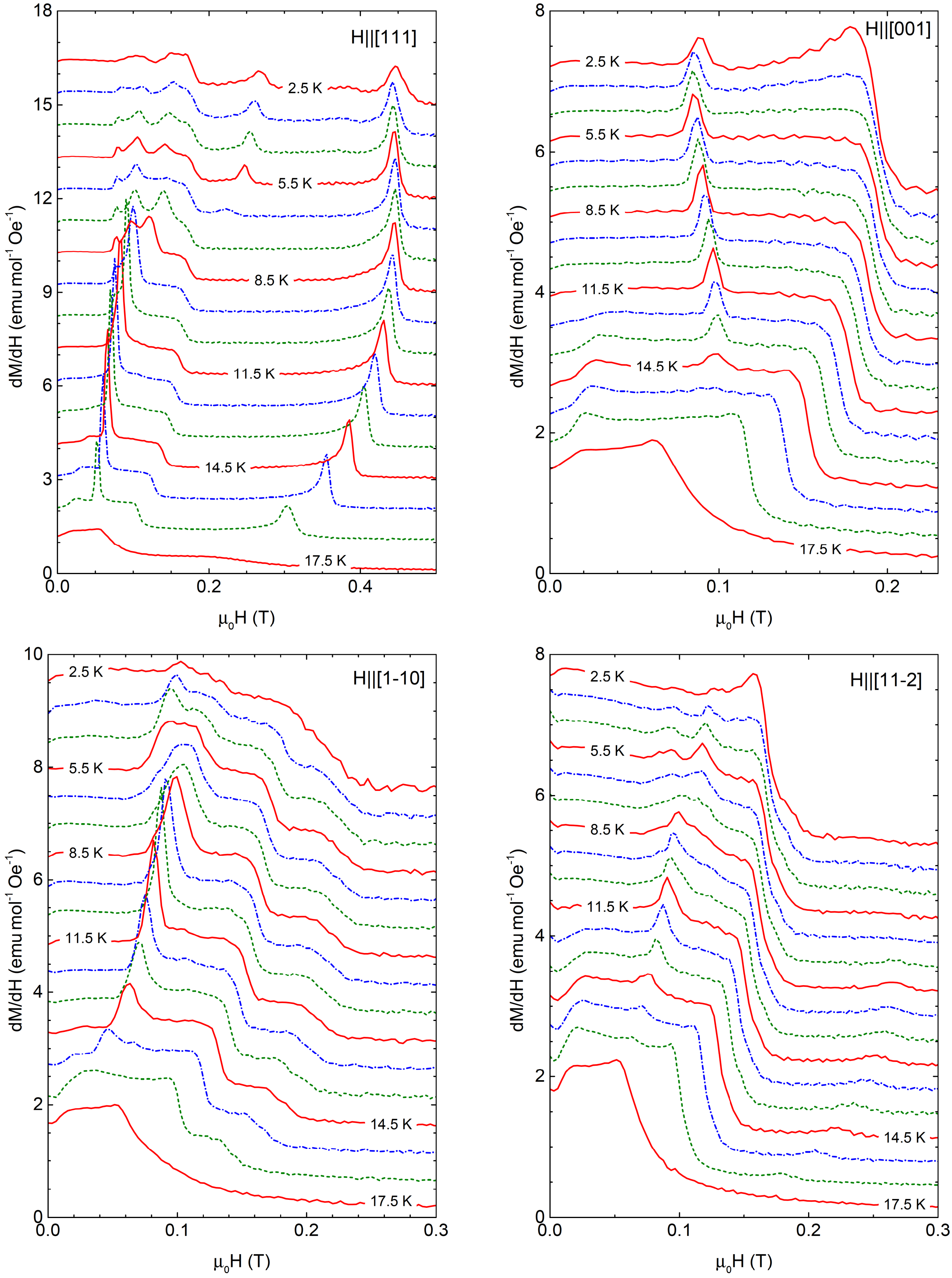

FIG. 1: | **Determination of magnetic phase boundaries in GaV_4Se_8.** Differential susceptibility, $\partial M/\partial H$ versus H, curves measured for four different orientations of the magnetic field, [111], [001], [1-10] and [11-2], at various temperatures. Curves are shifted vertically in proportion to the measurement temperature. Every second curve is omitted for clarity.
